# Perceptions of care quality and human rights in Italian outpatient facilities during and after the pandemic

**DOI:** 10.3389/fpubh.2026.1718407

**Published:** 2026-04-08

**Authors:** Elisa Cantone, Goce Kalcev, Massimo Tusconi, Alessandra Perra, Gabriele Finco, Clelia Madeddu, Laura Atzori, Elisabetta Cotti, Fabrizio Bert, Antonio Preti, Giuseppe La Torre, Mauro Carzedda, Orsola Marra, Francesco Tomaso Muscas, Viviana Forte, Stefano Lorrai, Caterina La Cascia, Laura Loli Dano, Mehmet Eskin, Fatma Charfi, Michela Atzeni, Enzo Tramontano, Giulia Cossu, Mauro Giovanni Carta

**Affiliations:** 1 PhD Program “Capacities Building for Global Health”, University of Cagliari, Cagliari, Italy; 2 Department of Medical Sciences and Public Health, University of Cagliari, Cagliari, Italy; 3 University Hospital of Cagliari, Cagliari, Italy; 4 Department of Surgical Sciences, University of Cagliari, Cagliari, Italy; 5 Department of Public Health and Pediatrics Sciences, University of Torino, Torino, Italy; 6 Department of Neurosciences, University of Torino, Torino, Italy; 7 Department of Public Health and Infectious Diseases, “Sapienza” University of Rome, Rome, Italy; 8 Biomedicine, Neuroscience, and Advanced Diagnostics, University of Palermo, Palermo, Italy; 9 Ryerson University, Toronto, ON, Canada; 10 Psychology Department, Koc University, Istanbul, Turkey; 11 Tunis El Manar University, Tunis, Tunisia; 12 Department of Life and Environmental Sciences, University of Cagliari, Cagliari, Italy; 13 Universidad Popular del Cesar, Valledupar, Colombia

**Keywords:** healthcare workers, job satisfaction, pandemic, patients, post-pandemic, respect for human rights, WWRR

## Abstract

**Background:**

This study investigated whether job satisfaction and perceptions of respect for human rights among healthcare workers and patients improved in the post-COVID period compared to during the pandemic in 2021.

**Methods:**

Conducted in October 2025 across five outpatient hospital units in Sardinia, Italy, this cross-sectional survey involved 97 healthcare professionals and 129 patients recruited voluntarily based on service requirements attendance. The “Well-Being at Work and Respect for Human Rights” questionnaire (WWRR) was used to assess job satisfaction, organizational climate, respect for human rights, and perceived adequacy of resources. Chi-square tests were used to compare categorical variables, and one-way ANOVA with Bonferroni correction was used to analyze mean WWRR scores.

**Results:**

Results indicated that, compared to 2021, healthcare workers’ scores remained largely unchanged, reflecting modest satisfaction and ongoing concerns about organizational factors, staffing, and resource availability. Conversely, patients reported significantly higher satisfaction, particularly in areas of care quality, organizational efficiency, and respect for rights, with notable improvements in organizational satisfaction since 2021 (*p* < 0.0001 for all comparisons). The greatest perception gap was observed in organizational aspects, where patient scores exceeded staff scores by nearly two points. Both groups continued to express dissatisfaction with resource adequacy. Additionally, staffing needs evolved, with increased demand for administrative support, security personnel, and multidisciplinary care providers. In 2025, compared to 2021, healthcare professionals reported a significantly greater need for administrative or HR managers (14.4% vs. 0%; *p* < 0.0001), security personnel (5.1% vs. 0.6%; *p* = 0.023), and an increase in all professional figures or multiple types of workers (14.4% vs. 0%; *p* < 0.0001). Similarly, user responses showed a higher perceived need for administrative or staff-managing professionals (8.5% vs. 0%; *p* < 0.0001) and occupational therapists, educators, or rehabilitation technicians (8.5% vs. 2.1%; *p* = 0.017).

**Conclusion:**

While patient trust in healthcare services has largely rebounded in the post-pandemic period, this masks ongoing challenges in restoring healthcare workers’ morale and satisfaction. Persistent systemic issues strain staff well-being and threaten the quality, efficiency, and sustainability of care. Bridging the gap between renewed patient confidence and the unresolved difficulties faced by healthcare workers must become a central priority for policymakers and administrators. By improving workplace conditions, fostering supportive organizational cultures, and investing in staff development and well-being, healthcare systems can secure a durable, post-crisis recovery.

## 1 Introduction

The recent COVID-19 pandemic has exposed and exacerbated organizational challenges and disruptions in delivering healthcare services worldwide (1). Many countries have experienced excess mortality due to cardiovascular (2) and cancer-related (3) causes. This was thought to be due to several factors, but primarily because people were less likely to seek treatment, particularly hospital care (4). Furthermore, as resources were redirected to combating the pandemic, treatment options became limited. In fact, the disruption of routine care due to the strain on healthcare systems during the COVID-19 pandemic has been well documented in both cardiology and oncology wards (5). These challenges affected the quality of healthcare delivered, the satisfaction of both users and healthcare workers, and the perception that the rights of both parties were not always respected (6). The aim of this study is to investigate, in a sample of healthcare workers from four outpatient departments of the University Hospital of Cagliari (Oncology and Pain Therapy, Dermatology, Cardiology and Endocrinology), whether job satisfaction and the perception of respect for the rights of healthcare staff and patients have improved compared to the findings of a similar study conducted in 2021 (6), during the height of the pandemic. The cited previous study found that healthcare professionals reported lower satisfaction with health services provided and perceived respect for their rights compared to users (6). Nevertheless, the study data suggest the presence of a degree of mutual trust between users and healthcare workers across all care facilities. In the four outpatient services included, the perceptions of organizational well-being and job satisfaction among healthcare workers, as well as users’ satisfaction with the care received, appear to be relatively high, though not at optimal levels. Notably, users’ satisfaction was equal to, and in some cases paradoxically higher than, that reported by healthcare workers. The data presented in this paper are significant as they derive from the first study to compare the perspectives of both users and healthcare workers. Moreover, the study was conducted after the first two waves of the pandemic, a period during which a heightened state of vulnerability and risk was assumed to affect all healthcare workers, including those not directly involved in COVID-19 care (6). Furthermore, satisfaction levels at work among professionals in these outpatient services were significantly lower than those reported by professionals working in mental health services (7). These results were interpreted as consequences of the difficulties affecting healthcare delivery (1) and the stress experienced by healthcare workers during the COVID-19 pandemic (8–10). In fact, the healthcare units analyzed in both the previously cited study and the present one were located within a hospital setting. Working conditions in hospitals during the COVID-19 pandemic limited interpersonal contact for health professionals due to fears of infecting family and friends. This placed them in a “siege-like” and more challenging position compared to those working in mental health care, which in Italy is primarily delivered through community-based services without reliance on psychiatric hospitals (7). It can be hypothesized that the community organization of mental health care is at the basis of a better perception of subjective well-being, organizational well-being, users’ satisfaction and respect for human rights by the staff of mental health services in Italy compared to the other specialized care providers that are still geographically and structurally linked to a hospital-centered model (7). Moreover, the previous international comparative research found mental health Italian professionals as those with the best scores at Well-Being at Work and Respect for Human Rights Questionnaire compared to mental health workers in other countries, who, unlike Italy, mainly work in hospital centered mental health facilities (11, 12).

The main objective of this study is to assess, in a group of healthcare workers from four outpatient departments at the University Hospital of Cagliari (Oncology and Pain Therapy, Dermatology, Cardiology, and Endocrinology), whether job satisfaction and the perception of respect for the rights of both healthcare staff and patients have improved compared to the results of a similar study conducted in 2021. The findings of this study will contribute to assessing whether there is a significant improvement in healthcare workers’ satisfaction levels in the post-crisis period, serving as a crucial indicator for the future strengthening of the healthcare system.

## 2 Materials and methods

### 2.1 Study design

This study adopted a cross-sectional comparison between healthcare workers and users, in line with previous research that investigated perceptions of quality of care, organizational climate, and respect for human rights in healthcare facilities (6). Diachronic analysis was applied to samples of healthcare workers and users during and after the pandemic. The study design, data collection, and reporting were performed in accordance with the STROBE cross-sectional reporting guidelines.

### 2.2 Study sample

A voluntary sample comprising health professionals working in five outpatient units (pain therapy, oncology, dermatology, endocrinology, and cardiology) of a single hospital in Sardinia, Italy, was recruited. These services were selected because they encompass diverse clinical settings with ongoing patient access and collaboration among multidisciplinary staff. This makes them appropriate for assessing post-pandemic perceptions of care quality and respect for patients’ rights. Participant recruitment took place between October 7 and October 14, 2025. The sample size was based on the number of eligible individuals available within the participating outpatient services during the specified data collection period, in order to replicate and compare the findings of a previous survey conducted in the same setting during the COVID-19 pandemic in 2021. Participants were recruited in person within the outpatient services of the University Hospital of Cagliari. Healthcare professionals working in the participating units were invited to take part in the survey during their work shifts, while patients were approached in the waiting areas of the same outpatient services during their scheduled visits. The questionnaires were administered in paper format and completed anonymously on site immediately after informed consent was obtained. No online distribution methods (e.g., email invitations, website links, or SMS invitations) were used. Participants were eligible for inclusion if they met the following criteria: (i) age ≥18 years (18 years + 1 day); (ii) either sex; (iii) provision of written informed consent; and (iv) active professional engagement in healthcare within one of the following categories: physicians, nurses, psychologists, healthcare assistants (OSS), biologists, social workers, rehabilitation professionals, educators, as well as postgraduate trainees in psychology and medical specialties. In accordance with the approved study protocol, participants were enrolled through voluntary recruitment based on service attendance; in total, 97 health professionals participated and were included in the present study sample. Two individuals (one medical doctor and one nurse) declined to participate due to time constraints, representing 2.06% of those approached and yielding a response rate of approximately 97.9%. All questionnaires were fully completed and included in the analysis; no cases were excluded due to missing data.

On the other hand, users involved were all patients receiving treatment at the same facilities. Exclusion criteria for users were: attending the facility for a first medical visit without prior experience, or experiencing a serious health crisis that would make completing the study instruments difficult or inadvisable given their condition. Users were recruited in the waiting areas of the same outpatient services during their scheduled visits and were invited to complete the questionnaire anonymously after informed consent. In total, 129 users participated and were included in the study sample. Three users were excluded based on these criteria, and none of the remaining users refused to participate, resulting in a 100% participation rate among eligible users approached.

### 2.3 Study tools

After signing a declaration of informed consent (approved by the ethics committee), both health professionals and users were asked to complete the following study questionnaires:

- A form designed to collect participants’ demographic information (age, gender, place of employment, and occupational role). To preserve anonymity, since cross-referencing the data could potentially lead to identification, less frequent professions (e.g., social worker, nutritionist, dentist, security agent) were grouped into a broader category. Similarly, no user diagnoses were recorded for the same reason. This approach was already adopted in the previously cited survey (6).
- The “Well-Being at Work and Respect for Human Rights Questionnaire” (WWRR) (11–13) is available in two versions: one for healthcare professionals and one for users. The WWRR was inspired by the World Health Organization’s Quality Rights project (14, 15). This project is grounded in the principles of the UN Convention on the Rights of Persons with Disabilities, placing respect for the rights of both users and staff at the core of healthcare facility quality assessments (16–19). A detailed description of the WWRR items has been provided in previous studies (11, 12), which demonstrated psychometric validity, internal consistency, and cross-cultural applicability in different healthcare settings and countries. The WWRR measures perceived respect for rights among both health workers and users, as well as the organizational climate within the care facility (or workplace for staff), and satisfaction with the care delivered (or with work conditions for staff). Items 1 to 5 are scored on a 1–6 Likert scale, where 1 indicates “Not satisfied at all” and 6 indicates “Completely satisfied.” Item 6 assesses the adequacy of resources in the care facility (or workplace), with responses coded on a reverse 1–5 Likert scale, where 1 means “Completely satisfied” and 5 means “Not satisfied at all.” Item 7 captures the interviewee’s perception of which type of health worker is lacking in the facility.

### 2.4 Statistical analysis

Data were analyzed using the Statistical Package for the Social Sciences (SPSS), version 29 (IBM Corp., 2021). Before conducting inferential tests, assumptions such as normal distribution were checked. Statistical analysis was performed by comparing categorical variables using the chi-square test with Yates correction applied when necessary. The mean ± standard deviation scores of items 1–6 of the WWRR were compared across groups (staff vs. users in 2025; staff 2025 vs. staff 2021; and users 2025 vs. users 2021) using one-way ANOVA with Bonferroni correction for multiple comparisons. No standardization for age and sex was applied in the comparison between staff and users, as the two recruitment methods reflected the respective populations. Therefore, the survey aimed to capture and compare the perspectives of these different populations as represented in the participating services.

Given the very low refusal rate and the in-person administration of the questionnaires, potential non-response bias and early–late response bias were considered minimal. In addition, common method bias was mitigated through anonymous participation and the use of a previously validated instrument (WWRR).

### 2.5 Ethical considerations

The study was approved by the Ethics Committee of the Region of Sardinia on September 18, 2025, under protocol number 25569. The study was carried out according to the Declaration of Helsinki and its revisions (20). Each participant signed an informed consent in which it was explained that the data would be collected in a database with anonymous records and each participant would be free to abandon the research at any moment if he/she want. Participants may direct any questions or requests for clarification regarding the study to the provided telephone number or e-mail address.

## 3 Results

Table 1 presents the comparison of demographic characteristics, including gender (male prevalence) and age (participants over 49 years old) among three groups: health workers in 2025 (used as the pivot sample) vs. health workers in 2021; users in 2025 vs. users in 2021; and health workers vs. users in 2025. In addition, the two samples of health workers were compared regarding the proportion of medical doctors versus other healthcare professionals.

**Table 1:** Socio-demographic characteristics of professionals and users (2025–2021).

Variable	Health workers 2025 (*N* = 97) *N* (%)	Health workers 2021 (*N* = 154) *N* (%)	Chi square	Users 2025 (*N* = 129)	Users 2021 (*N* = 142)	Chi square	2025 health workers vs. users
			*p*			*p*	Chi square
			OR (CI 95%)			OR (CI 95%)	*p*
							OR (CI 95%)
Gender (male)	29 (29.9)	61 (39.6)	2.411	46 (35.6)	52 (36.6)	0.027	0.31
			*p* = 0.118			*p* = 0.869	*p* = 0.583
			OR = 0.65 (0.4–1.1)			OR = 0.96 (0.6–1.6)	OR = 0.85 (0.5–1.5)
Age (≥49)	36 (37.1%)	65 (42.2)	0.642	110 (85.3%)	93 (65.5)	20.189	56.151
			*p* = 0.423			*p* < 0.0001	*p* < 0.0001
			OR = 0.81 (0.5–2.4)			OR = 3.67 (2.0–6.6)	OR = 0.10 (0.05–0.2)
Medical doctors	34 (35.0)	52 (33.3)	0.044	Not appropriate	Not appropriate	Not appropriate	Not appropriate
			*p* = 0.835				
			OR = 1.05 (0.6–1.8)				

No statistically significant differences emerged between the two diachronic samples of health professionals. Both samples showed a low prevalence of males and a low proportion of participants over 49 years old. The proportion of physicians was approximately one-third in both samples. Among users, the low frequency of males was similarly observed; however, a significantly higher proportion of individuals over 49 years was found in the 2025 user sample compared to 2021 (85.3% vs. 65.5%, *p* < 0.0001). Furthermore, the percentage of users over 49 years old in 2025 was significantly higher than that of health professionals in the same year (85.3% vs. 37.1%, *p* < 0.0001).

Table 2 presents the comparison of mean scores for items 1–6 of the WWRR questionnaire among health workers surveyed in 2025 (post-COVID-19 pandemic) and those surveyed in 2021 (during the COVID-19 pandemic). The 2021 data were collected from a sample drawn from the same population of health professionals during the pandemic period (6). No statistically significant differences were observed in any of the six items between the two time points. However, item 2 (“How much do you believe that the users of the service in which you work are satisfied?”) approached statistical significance, with a slightly higher mean score in 2025 (4.38 ± 0.96) compared to 2021 (4.09 ± 1.37; *p* = 0.066).

**Table 2:** WWRR item responses: healthcare professionals 2025 (post-COVID-19 pandemic) vs. 2021 (during the COVID-19 pandemic).

Question (Item)	Healthcare professionals 2025 (97)	Healthcare professionals 2021^a^ (154)	*F* (1, 249 df)	*p*
WWRR 1—How satisfied are you with your work? (users: of the services in which you are cared)?	4.19 ± 1.32	4.15 ± 1.06	0.071	0.791
WWRR 2—How much you believe that the users of the service in which you work are satisfied? (users: of the services in which you are cared)?	**4.38 ± 0.96**	**4.09 ± 1.37**	**3.405**	**0.066**
WWRR 3—How satisfied are you with the organizational aspects of your work/how your work is organized? (users: the work of the services in which you are cared)?	3.43 ± 1.38	3.24 ± 1.33	1.298	0.257
WWRR 4—To what extent do you believe that the human rights of the people who are cared for in your service are respected? (users: of the services in which you are cared)?	4.57 ± 1.35	4.53 ± 1.30	0.056	1.118
WWRR 5—To what extent do you believe that the human rights of the staff working in your service are respected? (users: of the services in which you are cared)?	4.07 ± 1.49	3.88 ± 1.48	0.992	0.320
WWRR 6—How do you evaluate the current state of care in your service/ward, with reference to resources? (users: of the services in which you are cared)?	3.00 ± 0.99	3.10 ± 0.94	0.656	0.419

a Reference number 6.

Table 3 presents the comparison of mean scores for WWRR items 1–6 between users and healthcare professionals surveyed in 2025. Across all six items, users reported significantly more positive perceptions than healthcare professionals (*p* < 0.0001 for all comparisons).

- *Item 1* (“How satisfied are you with the care you receive?”/“How satisfied are you with your work?”) showed users were more satisfied with the care they received than professionals were with their work (5.48 ± 0.92 vs. 4.19 ± 1.32).
- *Item 2* revealed that users were more optimistic in their belief that service users are satisfied with the care (5.13 ± 1.00 vs. 4.38 ± 0.96).
- *Item 3*, which assesses satisfaction with organizational aspects of the service, showed the largest gap between the two groups: users rated it much more favourably than professionals (5.20 ± 1.23 vs. 3.43 ± 1.38). Notably, this item had the lowest mean score among professionals across all six items.
- *Item 4* demonstrated that users had a better perception of respect for users’ human rights in the care facility (5.47 ± 1.07 vs. 4.57 ± 1.35).
- *Item 5* showed that users were also more optimistic about the respect for the human rights of healthcare professionals working in the center that provides the care (5.10 ± 1.12 vs. 4.07 ± 1.49).
- *Item 6* (which is coded in reverse, i.e., a low score means greater satisfaction) shows greater satisfaction with the resources in the service of users (2.35 ± 0.95 vs. 3.00 ± 0.99; *p* < 0.0001); however, since it is a scale of 1–5, the users’ responses would correspond to an average score of 3 if compared with the other scores; therefore, it is “still” the lowest score among users.

**Table 3:** WWRR item responses: healthcare professionals 2025 (post-COVID-19 pandemic) vs. users 2025 (post-COVID-19 pandemic).

Question (Item)	Healthcare professionals 2025 (97)	Users 2025 (129)	*F* (1,224 df)	*p*
WWRR 1—How satisfied are you with your work? (users: of the services in which you are cared)?	4.19 ± 1.32	5.48 ± 0.92	74.883	<0.0001
WWRR 2—How much do you believe that the users of the service in which you work are satisfied? (users: of the services in which you are cared)?	4.38 ± 0.96	5.13 ± 1.01	13.970	<0.0001
WWRR 3—How satisfied are you with the organizational aspects of your work/how your work is organized? (users: the work of the services in which you are cared)?	3.43 ± 1.38	5.20 ± 1.23	103.208	<0.0001
WWRR 4—To what extent do you believe that the human rights of the people who are cared for in your service are respected? (users: of the services in which you are cared)?	4.57 ± 1.35	5.47 ± 1.07	31.246	<0.0001
WWRR 5—To what extent do you believe that the human rights of the staff working in your service are respected? (users: of the services in which you are cared)?	4.07 ± 1.49	5.10 ± 1.12	35.210	<0.0001
WWRR 6—How do you evaluate the current state of care in your service/ward, with reference to resources? (users: of the services in which you are cared)?	3.00 ± 0.99	2.35 ± 0.95	24.999	<0.0001

Table 4 presents the comparison of mean scores for WWRR items 1–6 among users surveyed in 2025 (post-COVID-19 pandemic) and 2021 (during the pandemic). Overall, the data show a general trend toward more optimistic responses in 2025, with higher mean scores in all items except for Item 6, which is reverse-coded (lower values indicate greater satisfaction).

**Table 4:** Comparison of WWRR item scores among users: 2025 (post-COVID-19 pandemic) vs. 2021 (during the COVID-19 pandemic).

Question (Item)	Users 2025 (129)	Users 2021^a^ (142)	*F* (1,269 df)	*p*
WWRR 1—How satisfied are you with your work? (users: of the services in which you are cared)?	**5.48 ± 0.92**	**5.13 ± 1.26**	**6.705**	**0.010**
WWRR 2—How much do you believe that the users of the service in which you work are satisfied? (users: of the services in which you are cared)?	5.13 ± 1.01	4.97 ± 1.12	1.513	0.220
WWRR 3—How satisfied are you with the organizational aspects of your work/how your work is organized? (users: the work of the services in which you are cared)?	**5.20 ± 1.23**	**4.85 ± 1.36**	**4.901**	**0.028**
WWRR 4—To what extent do you believe that the human rights of the people who are cared for in your service are respected? (users: of the services in which you are cared)?	5.47 ± 1.07	5.37 ± 1.16	0.541	0.463
WWRR 5—To what extent do you believe that the human rights of the staff working in your service are respected? (users: of the services in which you are cared)?	5.10 ± 1.12	4.94 ± 1.08	1.432	0.232
WWRR 6—How do you evaluate the current state of care in your service/ward, with reference to resources? (users: of the services in which you are cared)?	2.35 ± 0.95	2.31 ± 0.96	0.119	0.731

a Reference number 6.

Two items showed statistically significant improvement in the post-pandemic period:

- *Item 1* (“How satisfied are you with the services in which you are cared for?”) increased from 5.13 ± 1.26 in 2021 to 5.48 ± 0.92 in 2025 (*p* = 0.010).
- *Item 3* (“How satisfied are you with the organizational aspects of the work of the services in which you are cared for?”) improved from 4.85 ± 1.36 in 2021 to 5.20 ± 1.23 in 2025 (*p* = 0.028).

Table 5 presents the comparison of mean scores at Item 7 of the WWRR questionnaire, which asks participants to indicate which professional profiles they believe should be more represented in the facility where they receive care (for users) or work (for healthcare professionals). Multiple responses were allowed. The table compares responses across three dimensions: healthcare professionals in 2025 vs. 2021; users in 2025 vs. 2021; and healthcare professionals vs. users in 2025.

**Table 5:** Responses to Item 7 of the WWRR questionnaire among the two samples of healthcare professionals (2025 and 2021) and users (2025 and 2021).

Professional category	Health workers 2025 (*N* = 97) *N* (%) [rank]	Health workers 2021^a^ (*N* = 154) *N* (%) [rank]	Chi square	Users 2025 (*N* = 129) *N* (%) [rank]	Users 2021^a^ (*N* = 142) *N* (%) [rank]	Chi square	2025 healthcare workers vs. users
			*p*			*p*	
			OR (CI 95%)			OR (CI 95%)	Chi square
							*p*
							OR (CI 95%)
Medical Doctors	20 (20.6) [II]	24 (15.6) [III]	1.043	50 (38.8) [I]	43 (30.3) [I]	2.155	8.523
			*p* = 0.307			*p* = 0.142	***p* = 0.004**
			OR = 1.41 (0.7–2.7)			OR = 1.46 (0.9–2.4)	OR = 0.41(0.2–0.7)
Nurses	28 (28.9) [I]	44 (28.6) [I]	0.003	39 (30.2) [II]	40 (28.2) [II]	0.139	0.050
			*p* = 0.960			*p* = 0.709	*p* = 0.824
			OR = 1.01 (0.6–1.8)			OR = 1.07 (0.6–1.9)	OR = 0.94 (0.5–1.7)
OSS—professional for personal care	13 (13.3) [VI]	36 (23.3) [II]	3.769	11 (8.5) [IV]	9 (6.3) [V]	0.474	1.386
			*p* = 0.052			*p* = 0.491	*p* = 0.239
			OR = 0.50 (0.2–1.0)			OR = 1.38 (0.5–3.4)	OR = 1.66 (0.7–3.9)
Psychologists	15 (15.5) [III]	21 (13.6) [IV]	0.162	15 (11.6) [III]	24 (16.9) [IV]	0.363	1.526
			*p* = 0.688			*p* = 0.547	*p* = 0.217
			OR = 1.16 (0.6–2.4)			OR = 0.80 (0.4–1.6)	OR = 0.65 (0.3–1.3)
Occupational therapists/educators/technicians of rehabilitation	5 (5.1) [VIII]	15 (9.7) [V]	1.787	11 (8.5) [IV]	3 (2.1) [VII]	5.677	0.501
			*p* = 0.191			***p* = 0.017**	*p* = 0.479
			OR = 0.50 (0.2–1.4)			OR = 4.32 (1.2 15.8)	OR = 0.54 (0.2–3.0)
Social workers	2 (2.1) [X]	4 (2.6) [VII]	0.001^b^	2 (1.5) [VII]	4 (2.8) [VI]	0.087^b^	0.001^b^
			*p* = 0.999			*p* = 0.768	1.00
						OR = 0.54 (0.1–3.0)	OR = 1.34 (0.1–9.7)
			OR = 0.79 (0.1–4.4)				
Administrative/staff managing	14 (14.4) [IV]	0 [IX]	23.540	11 (8.5) [IV]	0 [VIII]	12.663	1.963
			***p* < 0.0001**				*p* = 0.165
			OR = Inf (Not Calc)			***p* < 0.0001**	
							OR = 1.81 (0.8–4.2)
						OR = Inf (Not Calc)	
Staff security	5 (5.1%) [VII]	1 (0.6) [VIII]	5.177	1 (0.8) VIII	0 [VIII]	0.008^b^	4.109
			***p* = 0.023**			*p* = 0.929	***p* = 0.043**
						OR = Inf (Not Calc)	
			OR = 8.31 (1.0–72.3)				OR = 6.96 (0.8–60.5)
None needs to be incremented	7 (7.2) [VII]	9 (5.8) [VI]		0 [IX]	18 (12.7) [III]	16.102	9.607
						***p* < 0.0001**	***p* = 0.002**
						OR = 0 (Not Calc)	
							OR = Inf (Not Calc)
All need to be incremented or many kind of workers need to be incremented	14 (14.4) [IV]	0 [IX]	23.540	0 [IX]	0 [VIII]	0.000 *p* = 1	21.770
			***p* < 0.0001**				***p* < 0.0001**
			OR = Inf (Not Calc)				OR = Inf (Not Calc)

a Reference number 6.

b Yates correction.

In the responses from healthcare professionals in 2025 compared to those in 2021, a statistically significant increase was observed in the proportion of respondents who indicated a need for more administrative or human resources (HR) managers (14.4% vs. 0%; *p* < 0.0001). Additionally, there was a significant increase in the perceived need for more security personnel (5.1% vs. 0.6%; *p* = 0.023). In the responses from healthcare professionals in 2025 compared to those in 2021, a statistically significant increase was also observed in the proportion of respondents who indicated the need to increase all professional figures or many types of workers (14.4% vs. 0%; *p* < 0.0001). For the remaining professional categories, no statistically significant differences were found between the two time points. Nurses consistently emerged as the most frequently cited professional group in need of increased presence in both surveys.

When comparing user responses in 2025 to those in 2021, a greater proportion of users identified the need to increase administrative or staff managing professionals (8.5% vs. 0%; *p* < 0.0001) as well as occupational therapists, educators, or rehabilitation technicians (8.5% vs. 2.1%; *p* = 0.017). Notably, in 2025, no users responded with “None needs to be increased,” in contrast to 12.7% who gave this response in 2021 (*p* < 0.0001), suggesting a broader perceived need for strengthening staff resources in the post-COVID-19 period. In both years, medical doctors were the most frequently cited professional category needing increased presence, indicating a consistently high perceived value of this role among users. In the 2025 internal comparison between healthcare staff and users, professionals more frequently indicated the need to increase security staff (5.1% vs. 0.8%; *p* = 0.043), were more likely to respond that no category needed to be increased (7.2% vs. 0%; *p* = 0.002), and more often selected the response “all need to be incremented” or “many types of workers need to be incremented” (14.4% vs. 0%; *p* < 0.0001), compared to users.

## 4 Discussion

This study examined whether job satisfaction and perceptions of respect for the rights of healthcare staff and patients have improved in the post-pandemic period compared to the height of the COVID-19 pandemic. Overall, users reported high levels of satisfaction and perceived improvements in organizational aspects. In contrast, healthcare workers provided more modest evaluations, with no statistically significant changes observed in most WWRR items between 2021 and 2025. It is worth noting that the two samples of healthcare workers (from 2021 and 2025) remained largely consistent in terms of age, gender, and professional distribution (i.e., doctors vs. non-doctors). This stability was expected, as the turnover resulting from retirements and new hires over the four-year period likely balanced out demographic shifts. In contrast, the 2025 user sample included a significantly higher proportion of individuals over the age of 49, likely reflecting the tendency of older adults to avoid hospital visits during the pandemic due to fear of infection (21–24). This behaviour was influenced by the well-documented increased risk of severe outcomes from COVID-19 in this age category (25–29), which consequently led to reduced access to medical care for many older adults patients (30–33).

The consistently higher scores reported by users compared with healthcare staff suggest a persistent perception gap between service recipients and providers. This finding aligns with previous literature indicating that patients tend to evaluate healthcare more positively than professionals, likely due to differing reference points: patients assess care relative to their personal expectations, whereas professionals measure it against clinical and organizational standards (34–36). Additionally, improved accessibility to healthcare services post-pandemic, particularly for the older adults, may have further amplified this optimistic perception among users.

The largest discrepancy emerged in organizational satisfaction, with users’ evaluations significantly exceeding those of healthcare staff. This finding reinforces the idea that operational inefficiencies and workload pressures remain major concerns for healthcare professionals (9, 37). Notably, the question on organizational satisfaction (item 3 of the WWRR) revealed a substantial difference, nearly two points between staff and users in 2025. Additionally, changes in staff responses concerning the need to increase professionals for administrative and personnel management roles, which also rose among users, reflect a clear awareness of the ongoing crisis within healthcare workforce capacity. Dissatisfaction among healthcare workers may be further exacerbated by the burnout experienced during the COVID-19 era (38–42), which likely continues to exert long-term effects (43–50). This enduring strain appears to have a mutually reinforcing relationship with dissatisfaction related to staff shortages and inadequate resources (51–54). A particularly telling indicator of this discontent is the increased demand for security personnel, as reflected in responses to item 7 of the WWRR.

In the post-COVID era, both in Italy and globally, the perception of healthcare workers has shifted dramatically from being hailed as “heroes” during the early stages of the pandemic to being viewed as culprits and executioners in its later phases (55–58). This shift, coupled with the ongoing systemic crisis, has contributed to a marked increase in violence against healthcare professionals, raising significant concern among researchers and the public alike (59–68). From the user perspective, the notable post-pandemic improvement in satisfaction with care and organizational aspects likely reflects both the restoration of service availability and an increased appreciation for accessible care following the COVID-19 restrictions. However, satisfaction with resources remained comparatively low among both users and healthcare workers, suggesting persistent challenges related to infrastructure, staffing, or equipment. This findings is consistent with existing evidence of the structural crisis affecting the Italian national health system (69–72).

Responses to the staffing-needs question (Item 7) revealed shifting priorities among both healthcare professionals and users. Among professionals, there was a notable increase in the perceived need for administrative and HR managers, security personnel, and various categories of healthcare workers, reflecting heightened awareness of organizational and safety support requirements. Meanwhile, users expressed greater demand for occupational therapists and rehabilitation technicians, indicating growing recognition of the benefits of multidisciplinary care. The disappearance of the “none needs to be increased” response in 2025 suggests rising expectations for adequate staffing across both groups. These findings illustrate a partial recovery from the pandemic’s impact: while patient satisfaction has improved in some areas, healthcare workers continue to confront persistent organizational and resource challenges. Addressing these issues will require targeted interventions, such as workload redistribution, enhanced administrative support, and dedicated staff well-being programs, rather than simply restoring pre-pandemic operations.

## 5 Limitations of the study

This study has several noteworthy limitations that should be carefully considered when interpreting the results. First, participation was voluntary, which introduces the possibility that individuals who chose to participate may differ systematically from those who did not, potentially limiting the generalizability of the findings. Second, there was an absence of demographic standardization between groups, such as differences in age, gender, socioeconomic status, or cultural background, which may have acted as confounding variables and influenced the outcomes independently of the variables under study. Third, the study relied heavily on self-report measures, which, while practical, are inherently vulnerable to subjective biases such as social desirability, or individual interpretations of survey items. Collectively, these limitations highlight the need for caution in generalizing the findings and underscore the importance of future research employing more controlled designs, objective measures, and representative sampling strategies. Nevertheless, the study followed a structured sampling pattern based on predetermined data collection days. Combined with the very low refusal rate, this approach strengthens the representativeness of the samples and supports the reliability of the findings. Future research should prioritize the integration of longitudinal study designs alongside in-depth qualitative methodologies to more thoroughly investigate the complex, multifaceted factors that contribute to the ongoing and persistent perception gap between patients and healthcare staff. By capturing temporal changes and exploring detailed personal experiences, beliefs, and interactions over time, such research can provide a richer understanding of the underlying drivers of these perceptual discrepancies. Moreover, this approach would enable the development of evidence-based, contextually informed strategies and interventions that are specifically tailored to bridge the gap, enhance mutual understanding, and ultimately foster a more cohesive, patient-centered care environment in which the experiences of both patients and healthcare professionals are more closely aligned.

Based on the findings, it is recommended that healthcare organizations prioritize interventions targeting staff workload, administrative support, and well-being programs to address persistent dissatisfaction and burnout. Future research should explore longitudinal impacts of post-pandemic staffing and resource challenges, including strategies to bridge the perception gap between users and professionals. In education, curricula could emphasize resilience, multidisciplinary collaboration, and crisis management to better prepare healthcare workers for systemic pressures. Finally, management should focus on enhancing organizational efficiency, security, and infrastructure.

## 6 Conclusion

While patient trust in healthcare services appears to have largely rebounded in the post-pandemic period, this positive trend masks a deeper, more persistent challenge within healthcare systems: the ongoing recovery of healthcare workers’ morale and satisfaction. These systemic issues not only strain the well-being of healthcare workers but also threaten the quality, efficiency, and sustainability of care delivery over the long term. Bridging this divide between restored patient confidence and the unresolved challenges faced by healthcare staff must therefore become a central priority for healthcare policymakers and administrators. By proactively addressing workplace conditions, fostering supportive organizational cultures, and investing in the professional development and well-being of healthcare personnel, health systems can achieve a durable recovery in the post-crisis era.

## Data Availability

The datasets of this study will not be publicly available due to individual privacy rules.
